# “What about diet?” A qualitative study of cancer survivors' views on diet and cancer and their sources of information

**DOI:** 10.1111/ecc.12529

**Published:** 2016-06-28

**Authors:** R.J. Beeken, K. Williams, J. Wardle, H. Croker

**Affiliations:** ^1^ Department of Epidemiology and Public HealthHealth Behaviour Research CentreUniversity College LondonLondonUK

**Keywords:** beliefs, cancer survivorship, diet, information, knowledge, media

## Abstract

Given the abundance of misreporting about diet and cancer in the media and online, cancer survivors are at risk of misinformation. The aim of this study was to explore cancer survivors' beliefs about diet quality and cancer, the impact on their behaviour and sources of information. Semi‐structured interviews were conducted with adult cancer survivors in the United Kingdom who had been diagnosed with any cancer in adulthood and were not currently receiving treatment (*n* = 19). Interviews were analysed using Thematic Analysis. Emergent themes highlighted that participants were aware of diet affecting risk for the development of cancer, but were less clear about its role in recurrence. Nonetheless, their cancer diagnosis appeared to be a prompt for dietary change; predominantly to promote general health. Changes were generally consistent with healthy eating recommendations, although dietary supplements and other non‐evidence‐based actions were mentioned. Participants reported that they had not generally received professional advice about diet and were keen to know more, but were often unsure about information from other sources. The views of our participants suggest cancer survivors would welcome guidance from health professionals. Advice that provides clear recommendations, and which emphasises the benefits of healthy eating for overall well‐being, may be particularly well‐received.

## 1 Introduction

With increasing numbers of people surviving cancer due to earlier detection and better treatments (Maddams, Utley, & Møller, 2012), there is growing interest in the potential of lifestyle factors, such as diet, as a way of reducing the late and long‐term effects of cancer. There is good evidence that a healthy diet (plant‐based with limited intake of high calorie foods, red meat and processed meats) can help prevent cancer (Cancer Research UK, 2015; WCRF and AICR, 2007). Observational studies have shown that a low‐fat/high‐fibre diet is protective against progression of breast, colorectal and prostate cancers, and there is evidence of an increased risk of breast cancer recurrence from consuming a “Western diet” (Kroenke, Fung, Hu, & Holmes, 2005; Patterson, Cadmus, Emond, & Pierce, 2010).

The mechanisms linking dietary fat intake with cancer outcomes are not well understood but are thought to be related to sex hormones such as oestrogen. For example, dietary fat intake has been shown to increase levels of oestrogen in the blood, which may promote the development of breast cancer in women (Wu, Pike, & Stram, 1999). Fibre is thought to be protective against colorectal cancer because it dilutes faecal contents, increases stool weight and decreases gastrointestinal transit time, potentially reducing exposure to carcinogens (WCRF & AICR, 2007). Dietary fibre may also lead to the production of short‐chain fatty acids in the colon, which have been shown to promote apoptosis, potentially reducing the risk of cancer developing (WCRF & AICR, 2011). On the other hand, intervention studies suggest that diet may influence outcomes indirectly via its role in energy balance (Chlebowski et al., 2006; Pierce et al., 2007). However, cancer survivors (defined as “all people who are living with a diagnosis of cancer, and those who have recovered from the disease” [WCRF & AICR, 2007]) are also at increased risk of second primary cancers as well as other chronic conditions such as diabetes, osteoporosis and cardiovascular disease (Brown, Brauner, & Minnotte, 1993; Travis et al., 2006), and diet is an important modifiable factor that could reduce these risks, thereby promoting their long‐term health. Dietary change may also impact quality of life in cancer survivors, particularly for those diagnosed with prostate, breast and colorectal cancer (Kassianos, Raats, Gage, & Peacock, 2015).

Previous studies suggest that few cancer survivors attribute the development of their cancer to a poor diet (Willcox, Stewart, & Sitas, 2011). However, little is known about whether cancer survivors believe diet to be important for their long‐term health, post‐diagnosis. A recent survey of 3,300 colorectal cancer survivors found that over 20% would like more advice on diet and lifestyle, suggesting that many do not feel sufficiently informed in this area (Department of Health‐Quality Health., 2012). Some survivors may want information about diet because of specific nutritional needs or side effects post‐treatment, whereas others may want more information for their general health or to prevent recurrence. Previous studies with breast cancer survivors have found that some are aware that diet may play a role in reducing cancer recurrence (Burris, Jacobsen, Loftus, & Andrykowski, 2012; Weiner, Jordan, Thompson, & Fink, 2010), but survivors are often unsure what constitutes a healthy diet (Maley, Warren, & Devine, 2013). Some studies have shown that cancer survivors report trying to eat a healthy diet following their diagnosis (Lim, Gonzalez, Wang‐Letzkus, Baik, & Ashing‐Giwa, 2013; Maskarinec, Murphy, Shumay, & Kakai, 2001; Meraviglia & Stuifbergen, 2011; Satia, Walsh, & Pruthi, 2009; Wang & Chung, 2012), however it is unclear what guides these dietary choices.

Many organisations have lifestyle guidelines for cancer prevention (Kushi et al., 2012; NHS Choices, 2014; WCRF and AICR, 2007), but recommendations for cancer survivors are more limited because of insufficient evidence linking diet directly to cancer outcomes. Those that do exist therefore either refer to guidelines for cancer prevention (WCRF and AICR, 2007) or focus more on acute health and psychosocial outcomes or nutritional needs as a consequence of treatment, rather than long‐term survival (Schmitz et al., 2010). Surveys with health professionals suggest that few discuss lifestyle factors, including diet, with their cancer patients (Daley, Bowden, Rea, Billingham, & Carmicheal, 2008; Macmillan Cancer Support/ICM, 2011). Insufficient professional advice coupled with a desire for information may lead some cancer survivors to seek out information about diet themselves. This was found in a recent qualitative study of colorectal cancer survivors in the United Kingdom, where several people reported actively try to seek out further information about lifestyle factors such as diet (Anderson, Steele, & Coyle, 2013).

Active information‐seeking from media sources has been linked to increased fruit and vegetable consumption among colorectal cancer survivors (Lewis et al., 2012), and exposure to health news has been shown to increase knowledge about dietary cancer risks (Stryker, Moriarty, & Jensen, 2008). However, when searching in popular media or online, cancer survivors are likely to encounter a wealth of information, not all of which will be reliable and accurate. There is an abundance of media misreporting of the dietary factors that are linked to cancer risk (Goldacre, 2009) that could be misleading to patients, particularly if they believe the sources to be trustworthy. Previous studies have demonstrated that survivors do not rate media sources all that highly for general information about their disease and treatment (Chen & Siu, 2001), although one study found that those who use the Internet believe this to be a high‐quality source (Mills & Davidson, 2002). However, these studies did not explore survivors' use of the media for information about diet and were conducted some time ago. Determining cancer survivors' sources of information about diet and cancer will help understand why they hold particular beliefs about these factors.

Given that little is known about survivors' beliefs about the importance of diet post‐diagnosis and what guides dietary choices post‐diagnosis, a qualitative methodology was chosen to explore this issue. Qualitative research enables us to capture a range of views and to explore why those views are held. Although there are many benefits of quantitative methodologies, a qualitative study enables an in‐depth exploration of cancer survivors' beliefs about the role of diet in their long‐term health and helps us to better understand the sources behind their beliefs and dietary choices.

This study therefore aimed to explore, with a qualitative methodology, cancer survivors' beliefs about the role of diet in their long‐term health and survival, and their sources of information. This could ultimately inform the provision of evidence‐based dietary information to cancer survivors, and the development of effective dietary interventions.

## 2 Methods

### 2.1 Participants and recruitment

This was a qualitative interview study with adult cancer survivors (age ≥18 years) living in the United Kingdom, who had been diagnosed with any cancer during adulthood and were not currently receiving treatment for cancer. Because there are few tailored dietary recommendations for survivors, and we were interested in beliefs about the benefits of diet for long‐term health and survival in general, as opposed to nutritional needs specific to certain cancers/treatments, we sought to recruit a range of survivors. This also meant we would be representing a wide range of views, applicable to the wider survivorship population as opposed to focusing on a more specific group. Interviews were chosen over focus groups as we were interested in hearing about patients' individual beliefs and experiences, rather than determining a group consensus. We did not want individuals' unique beliefs and experiences to be influenced by group discussions or concerns that others might view their beliefs to be “incorrect”. Telephone interviews also encouraged individuals to take part that might have otherwise been put off by a lack of flexibility around time (e.g. because of work commitments) and location (e.g. because of distance). A qualitative methodology was chosen because we were not seeking to test a hypothesis, but rather to obtain a rich source of information to better understand the rationale behind dietary beliefs and changes in this population (Holliday, 2010).

The study was advertised via an advert on Cancer Research UK's “Cancer Chat” online forum (Cancer Research UK, 2014) and by posters and flyers displayed in the University College Hospital Macmillan Cancer Centre. Potential participants were asked to contact the study team by telephone or email to check eligibility, and a follow‐up telephone call was arranged for those making contact by email. During this telephone call, information was given about the study with an opportunity to ask questions. An interview was then arranged for those interested in taking part, either face‐to‐face (at the University) or over the telephone, depending on the participant's preference. A study information sheet, consent form and brief socio‐demographic questionnaire were mailed for completion before the interview took place. We aimed to recruit until it was felt that saturation had been reached. In line with other qualitative studies in similar groups, we anticipated that approximately 15 participants would be required for this to be the case (Meraviglia & Stuifbergen, 2011; Thewes, Butow, Girgis, & Pendlebury, 2004). Ethics approval was granted by the University College London Research Ethics Committee, reference 0793/004.

### 2.2 Data collection

Socio‐demographic questions covered gender, age, marital status, education and employment. It also included a check question about their cancer diagnosis (“Have you ever been diagnosed with cancer”), the primary cancer site (“If yes, which type”) and the date of diagnosis (“When were you diagnosed”).

**Figure 1:**
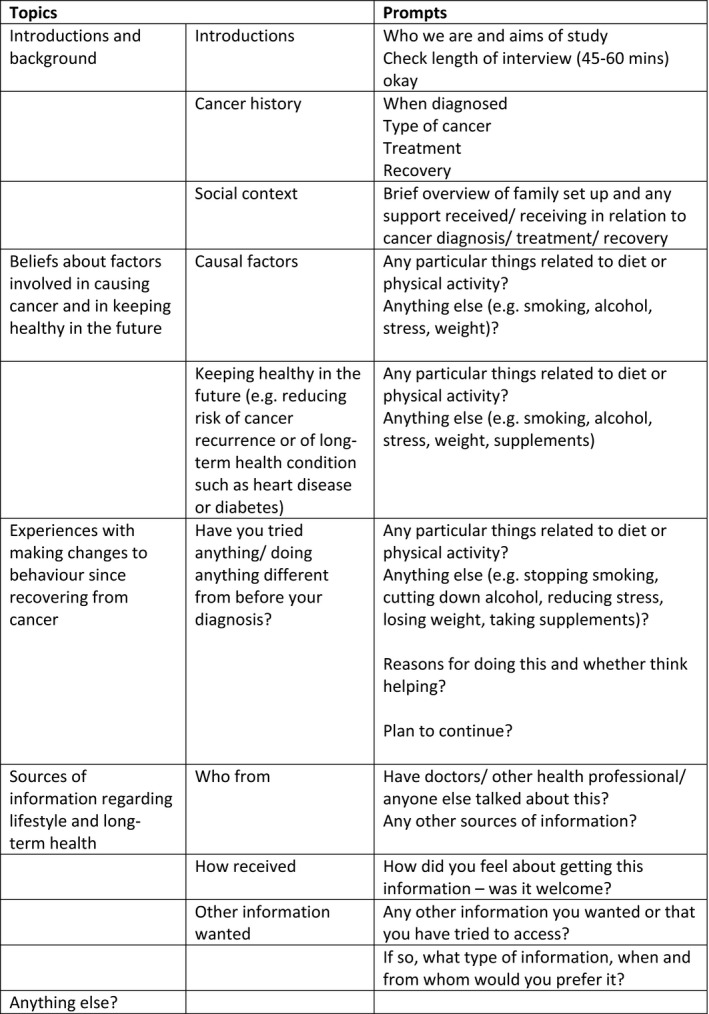
Topic guide for qualitative interviews

Semi‐structured interviews were carried out by three female researchers (KW, HC and RB) between March and July 2013. Interviews lasted approximately 1 hr, and were recorded and transcribed verbatim. A topic guide (Figure 1) was developed by HC, KW and RB to guide the interviews and consisted of a series of open questions covering beliefs about the relationship between diet and cancer, sources of information and changes to diet following cancer diagnosis. This was part of a broader interview that also covered participants' views about other lifestyle factors and cancer. Interviewers were trained to have minimal verbal input and prompt only when appropriate (Oppenheim, 1992). The topic guide was piloted with two participants whose data were included because no substantial changes were required.

### 2.3 Analysis

Data were analysed using Thematic Analysis, a qualitative method for identifying, analysing and reporting themes (Braun & Clarke, 2006). Thematic analysis was chosen to provide a rich description of the data, and to identify themes at an explicit level using a realist approach (Braun & Clarke, 2006). The first three transcripts were reviewed independently by three researchers (KW, HC and RB) who each generated an initial list of codes. These lists were then amended and refined through discussion between the researchers until a single list was agreed. A researcher (KW) entered the list of codes into NVivo version 10 (QSR International Pty Ltd, 2012) and coded all the transcripts, with codes added to the list where necessary. A random selection of transcripts (*n* = 5) were coded by a second researcher (HC) to check for reliability. Inter‐rater reliability for the coding was generally high (>.7) with any discrepancies discussed and resolved in discussion. Once the coding had been agreed, KW and RB reviewed the coded transcripts to search for common themes. These themes were reviewed and refined, named and each given a written description.

## 3 Results

### 3.1 Participants

Twenty‐four cancer survivors made contact having seen an advert for the study. Of these, two were not eligible because they lived abroad, two had contacted us about issues unrelated to our study (they were referred to the Cancer Research UK nurse help‐lines) and one did not respond to our attempts to contact them back. Nineteen interviews were conducted with 11 women and 8 men, aged between 24 and 77 years (Table 1); 5 face‐to‐face and 14 by telephone. All interviews were conducted with only the participant and interviewer present. Of the 19, 7 were recruited via the online forum and 12 were recruited through flyers. After the target number of 15 interviews was achieved, the authors discussed the themes emerging and whether saturation had been reached (Morse, 2000). Although it appeared that saturation was reached at this point, a further four interviews were conducted to confirm this. All participants described their ethnicity as White British, the majority were married (68%) and half were working in some capacity (53%). Educational attainment varied although the majority (58%) had a higher education qualification. Breast cancer was the most common diagnosis (37%) and the majority of participants had been diagnosed in the past 5 years (63%).

**Table 1:** Socio‐demographic and health characteristics

Socio‐demographic details	Total sample (*n* = 19)
Gender: *n* (%)	
Male	8 (42.1)
Female	11 (57.9)
Age (years): mean ± SD (range)	59 ± 13.11 (24–77)
Ethnicity: *n* (%)	
White British	19 (100.0)
Marital status: *n* (%)	
Single/never married	2 (10.5)
Married/living with partner	13 (68.4)
Married separated from partner	1 (5.3)
Divorced	3 (15.8)
Highest educational status: *n* (%)	
Degree or higher degree	9 (47.4)
Higher education below degree	2 (10.5)
Secondary school qualifications	5 (26.3)
No formal qualifications	1 (5.3)
Other	2 (10.5)
Employment status: *n* (%)	
Employed full time	5 (26.3)
Employed part time	2 (10.5)
Self‐employed	3 (15.8)
Retired	8 (42.1)
Disabled or too ill to work	1 (5.3)
Cancer diagnosis^a^: *n* (%)	
Breast	7 (36.8)
Colorectal	1 (5.3)
Prostate	1 (5.3)
Lung	1 (5.3)
Thyroid	2 (10.5)
Non‐Hodgkin lymphoma	3 (15.8)
Hodgkin lymphoma (Hodgkin disease)	1 (5.3)
Testicular	1 (5.3)
Bladder	1 (5.3)
Melanoma	2 (10.5)
Neuroendocrine tumour (NET)	1 (5.3)
Date of diagnosis: *n* (%)	
<5 years ago	12 (63.2)
5–10 years ago	4 (21.1)
11–20 years ago	2 (10.5)
>20 years ago	1 (5.3)

a Total comes to >100% as two people had been diagnosed with more than one type of cancer.

### 3.2 Themes

A number of themes emerged, which were as follows: (1) diet is a potential cause of cancer development, (2) diet is important for long‐term health, (3) a cancer diagnosis prompts dietary change and (4) a desire for more information about diet post‐diagnosis. There were no obvious differences in responses by cancer type, age or gender, so results are presented from the whole sample.

#### 3.2.1 Diet is a potential cause of cancer development

Participants described how they had tried to understand what might have caused their cancer: “for me it was like, well where did this come from, what's caused it?” (101, male, 60 years, non‐Hodgkin lymphoma), “Once I got the cancer it was like, ‘Ok, you have to find a reason for this’” (105, female, 51 years, breast cancer). This had led them to question if diet had played a role: “I've thought of food – is there food I am eating what's causing this?” (110, female, 51 years, breast and bladder cancer), “I think it's absolutely fascinating to know whether it is partly our diet” (103, female, 62 years, breast cancer and non‐Hodgkin lymphoma), “I honestly don't think I could have been doing anything wrong, apart from possibly something to do with my diet” (105, female, 51 years, breast cancer), “It's just that so many people these days, sadly, are getting cancer and I don't know whether it's the water or whether it's the food they eat” (114, female, 74 years, breast cancer).

Participants mentioned specific foods that they thought may contribute to the development of cancer. Occasionally, this related to the development of their own cancer: “It could be the result of eating too many crisps…I'm a bit of a crispaholic” (101, male, 60 years, non‐Hodgkin lymphoma), but more often it was discussed in relation to the onset of other types of cancer, or cancer in other people: “If you eat lots of fatty foods you're going to get, I don't know, some sort of cancer, diabetes, maybe, but, erm, that wasn't the case when it came to mine” (108, male, 24 years, NET), “I think additives are a danger, MSG and all this, I see it as a health danger but whether it can cause cancer or other conditions, I don't know” (116, male, 68 years, lung cancer). When participants did mention specific foods in relation to causing cancer, there were generally accurate beliefs. For example, red meat and burnt food were described as potential causal factors: “I sometimes think that red meat causes possibly bowel cancer” (102, male, 38 years, Hodgkin disease), “When the barbecue's black, that seems to be a big no‐no, is it carcinogenic or something, and I think the cancer develops on it or something” (105, female, 51 years, breast cancer). Others mentioned that fibre may help prevent cancer: “Certain nuts, apparently the high fibre in it's supposed to help stop you getting the cancer” (104, male, 69 years, prostate cancer), “I think if you have the right diet and plenty of roughage, everything is pushed out on a regular basis, but if it sits in there three or four days, this can be a contributing factor to the cancers growing” (109, male, 77 years, colon cancer).

#### 3.2.2 Diet is important for long‐term health

Participants talked about dietary factors that they thought might influence their long‐term health. Generally, they did not have strong beliefs about specific dietary components that could prevent recurrence, although they sometimes mentioned foods in relation to having had cancer: “I read that if you have a carcinoid tumour in your body, still, you need to avoid…spicy food such as curries” (108, male, 24 years, NET), “Like [for] bowel cancer, there are certain foods you are recommended to try and avoid. I think red meat is one” (104, male, 69 years, prostate cancer). More frequently they expressed general beliefs about specific foods that are healthy: “eating tomatoes, apparently, is supposed to be good for you, and nuts, tomatoes, anything, apparently, red‐coloured is supposed to help” (104, male, 69 years, prostate cancer), “Plenty of green veggies, i.e. broccoli and greens and things like that” (114, female, 74 years, breast cancer) or unhealthy: “my understanding is that white flour and sugar are kind of poison to your body” (105, female, 51 years, breast cancer). Methods of cooking that they believed were bad for them were also mentioned: “Just the fact that the way they're manufactured, the stuff's not fresh, it's not getting to you until it's been through all these processes….and it's kept in these polystyrene‐type dishes and stuff which you stick in the microwave or stick in the oven” (105, female, 51 years, breast cancer). Participants emphasised the importance of a balanced diet: “I believe you should have a little bit of everything. I am not one of these who think fruits and vegetables are going to change my life” (110, female, 51 years, breast and bladder cancer), “I think everything in moderation is the way” (116, male, 68 years, lung cancer).

Participants mentioned dietary supplements and views were polarised. Some believed that they were good for their health: “Selenium is very good for you” (114, female, 74 years, breast cancer), “manuka honey…it's meant to have antibacterial” (112, female, 69 years, non‐Hodgkin lymphoma), “magnesium…that's good for the bones” (114, female, 74 years, breast cancer), although they did not specifically mention them in relation to cancer cure or prevention of recurrence. In contrast, others believed dietary supplements could be harmful to health and even cause cancer: “there are some supplements that will give you cancer” (107, male, 50 years, melanoma), “I fundamentally disagree with them [supplements]. I am a pharmacist's daughter and just think it's all rubbish” (113, female, 47 years, thyroid cancer). Those who expressed negative attitudes towards supplements in this sample were from more academic backgrounds or had family members who worked in healthcare.

#### 3.2.3 A cancer diagnosis is a prompt for dietary change

Although participants were often aware of the benefits of a healthy balanced diet, it was not something that they had necessarily paid attention to until they were diagnosed with cancer: “I never really read up on [lifestyle] before…maybe I did and I just ignored it because we were all fine…then once I got the cancer…all the things that you used to do that they're saying are bad for you, you're trying to cut out” (105, female, 51 years, breast cancer). Participants had similar stories about how their cancer diagnosis had prompted them to make changes to their diet: “I have really, really looked at my diet since I was diagnosed with a lymphoma” (103, female, 62 years, breast cancer and non‐Hodgkin lymphoma), “you become acutely sensitised to anything cancer‐related….anything carcinogenic…you become really tuned into in terms of foodstuffs” (101, male, 60 years, non‐Hodgkin lymphoma). Participants did occasionally mention that they had made dietary changes to avoid cancer recurrence: “I read that this has more chance of coming back, then I have to cut out the only things that I can cut out now. I can't stop smoking because I never did, and I can't stop alcohol because I don't. So the only thing I've got to work on is my diet” (105, female, 51 years, breast cancer). However, they seemed to be more concerned about their long‐term health in general and wanted to give themselves the best chance at living a healthy life having survived their cancer diagnosis: “I think I'm probably more worried about [high blood pressure] than I am about getting cancer again” (115, female, 63 years, breast cancer), “I just felt that [dietary changes] would be better for my health” (114, female, 74 years, breast cancer). One participant also mentioned that weight management was a factor in their dietary choices: “If I am being honest, we did it [eat more healthily] more as part of the weight‐loss plan” (106, female, 50 years, breast cancer).

For these participants, eating a healthy balanced diet involved eating more of specific healthy foods, typically more fruit and vegetables: “I eat a lot more fruit than I ever did” (102, male, 38 years, Hodgkin disease). Some mentioned that they try to buy organic foods whenever they could as they believed this was better for them: “I try to go as organic as I can” (105, female, 51 years, breast cancer), “I am also into the organic lentils, sprouts and organic… you know, all better food, much better quality of food” (111, female, 63 years, thyroid cancer). Participants also talked about how they tried to avoid or cut down on particular unhealthy foods. They mentioned a range of foods but these were typically fatty, sugary foods and processed meat: “Cutting down on fatty food, I've reduced my intake of crisps” (101, male, 60 years, non‐Hodgkin lymphoma) and “Red meat, definitely, was reduced” (103, female, 62 years, breast cancer and non‐Hodgkin lymphoma), “I used to eat biscuits and cakes, cakes for breakfast, loved it, always loved cake for breakfast but I haven't had cake for ages, haven't had cake for ages. I might have an occasional biscuit but very rarely. So my diet has changed radically, as has my life” (111, female, 63 years, thyroid cancer). Others emphasised that they just tried to eat healthily and be sensible rather than following a particular diet or eating specific foods: “Just an ordinary, really healthy, sensible diet” (119, female, 67 years, melanoma).

Participants mentioned taking dietary supplements to benefit their health. Again the logic seemed to be about general health rather than a particular anti‐cancer property: “I have been taking supplements for years – magnesium, because that's good for the bones, selenium, as I said, vitamin C, I take that, and also I take a vitamin B which is very good” (114, female, 74 years, breast cancer), “I have a high dose of cod liver oil” (116, male, 68 years, lung cancer), “I take multivitamins and minerals every day” (108, male, 24 years, NET). For the most part, no explicit reasons for taking them were given and participants did not necessarily report any awareness of what they should/should not be doing. One participant said: “Selenium is supposed to be prevention from cancer” (114, female, 74 years, breast cancer) and therefore reported taking it regularly although she was unclear where this information had come from. In contrast, others mentioned that they avoided supplements. For some this was because they had been directed to for treatment reasons: “I've steered clear of all of them [supplements] because of the medication that I'm on” (115, female, 63 years, breast cancer), “I don't take supplements” (117, male, 65 years, testicular cancer). Some participants cited that their reason for avoiding supplements was because they preferred to get their vitamins and minerals from their diet: “It was more focused on…trying to increase my vitamins level naturally as opposed to taking supplements” (103, female, 62 years, breast cancer and non‐Hodgkin lymphoma), “I don't take pills very often. No, nothing. Just an ordinary, really healthy, sensible diet” (119, female, 67 years, melanoma).

#### 3.2.4 A desire for more information about diet post‐diagnosis

Participants were positive about the idea of getting dietary information, but did not generally recall receiving any from a health professional or had received only basic information or advice about lifestyle: “I didn't really get any advice about that…if anything, it was just try and eat a well‐balanced diet” (110, female, 51 years, breast and bladder cancer), “Well, shamefully, I wasn't given much information” (111, female, 63 years, thyroid cancer), “All I got, as I said, was a one‐off letter with a piece of paper in from the dietitian, saying how I can help to restore, recover and boost my phosphate levels and above, which is a very, very finely focused view of one aspect, one tiny aspect, of recovery from cancer” (101, male, 60 years, non‐Hodgkin lymphoma). It was reported that they had asked for dietary information: “I sort of said to my consultant, ‘What about diet?’” (103, female, 62 years, breast cancer and non‐Hodgkin lymphoma), and “should I be doing anything about my diet or anything while I'm doing this?” (105, female, 51 years, breast cancer). Some had even paid to see a health professional privately because they were not given sufficient information: “the other private appointment was the dietitian because there was nothing at the hospital for me” (105, female, 51 years, breast cancer). When participants had received advice from health professionals, this was not always consistent and sometimes added to their confusion about what they should do: “it was suggested by my breast care nurse that selenium might be a suitable supplement to take and to take it with vitamin A, C and E, as a combo…my current consultant doesn't seem to favour supplements” (103, female, 62 years, breast cancer and non‐Hodgkin lymphoma).

As professional advice seemed to be lacking, participants mentioned that they had researched information about lifestyle themselves. Participants reported seeking advice from cancer charities, and finding this helpful: “the information I got was the very, very good [charity name] booklets” (103, female, 62 years, breast cancer and non‐Hodgkin lymphoma), “there were lots of booklets on all sorts of things – living with cancer, the emotional aspects, the travel insurance, diet, all sorts of things” (112, female, 69 years, non‐Hodgkin lymphoma), “I went to [charity name] for most of my literature” (106, female, 50 years, breast cancer), “I phoned, once, …and they were fairly helpful” (111, female, 63 years, thyroid cancer). One participant also mentioned contacting a local organisation: “I phoned an organisation in [location] to ask about diet because this has been my problem; I don't know what's good and what's bad and whatever” (111, female, 63 years, thyroid cancer).

Survivors had also used the Internet for their research: “I went onto the Internet and found a few things. I just put in ‘anti‐cancer foods’ and got what came up” (105, female, 51 years, breast cancer), “I saw on the Internet, someone suggested a book”, “the Internet for hours and hours and hours, and printing off and printing off…they gave me a website to have a look at and I had a look at it, a thyroid cancer site. I've looked at all of them” (111, female, 63 years, thyroid cancer). Participants mentioned online charity forums as a source of information about lifestyle: “you get a lot of people with lots of ideas and suggestions” (101, male, 60 years, non‐Hodgkin lymphoma). However, participants said that they had difficulty sifting the reliable information from the wealth of nonsense online: “there is so much information and so many claims and counter‐claims, some good‐hearted or good‐willed, some just out to make money and some just plain scams that it's just impossible to tell one from another” (101, male, 60 years, non‐Hodgkin lymphoma), “when I was first diagnosed I went on a heck of a lot of different sites…I found some of them are downright misleading” (104, male, 69 years, prostate cancer), “the worst place of all is online…there are a lot of deliberately misinforming websites…go on any cancer patients' board…people will be promoting the vitamin‐type supplements. And then there's the magic fruit…, the noni and soursop…it's all claptrap” (107, male, 50 years, melanoma). One participant talked about how he had tried to clarify online information by reading research papers: “I read the secondary sources in the cases where there seemed to be something in it. I had a look at the primary sources…it was all groundless” (107, male, 50 years, melanoma).

In addition to doing their own research, participants mentioned obtaining information about lifestyle incidentally from the media: “I keep an eye on reports and media” (116, male, 68 years, lung cancer), “I get it by reading the paper” (117, male, 65 years, testicular cancer), “if there's an article in the newspaper, I'll read that, on cancer prevention” (118, female, 64 years, breast cancer), “you pick things up in the press” (115, female, 63 years, breast cancer). Participants also cited ‘facts’ about diet but could not recall where they had obtained particular information: “It's a well‐known fact [that supplements such as selenium and green vegetables are good for you]” (114, female, 74 years, breast cancer).

## 4 Discussion

This study aimed to explore cancer survivors' beliefs about the role of diet quality in cancer and to understand their sources of information. The results suggest that survivors are broadly positive about eating healthily, and participants reported making, or at least trying to make, some changes following their cancer diagnosis. Some specific foods and nutrients were mentioned as healthy (e.g. nuts, tomatoes, red foods, green vegetables, selenium, manuka honey) or unhealthy (e.g. red and processed meat, white flour, sugar, spicy food, processed food) but on the whole, participants perceived a healthy balanced diet as more important than specific foods or supplements. Although cancer was often a prompt for addressing lifestyle change, a healthy diet after diagnosis tended to be based on the belief that this was good for general health rather than specifically connected to cancer outcomes. Where diet was discussed specifically in relation to cancer, this was most often in connection with causing cancer as opposed to a role in cancer recurrence. However, in line with previous studies (Wold, Byers, Crane, & Ahnen, 2005), participants did not attribute the development of their own cancer to diet. Diet was cited as an important risk factor for cancer in general and in relation to other people rather than themselves. Participants reported not receiving dietary information from a health professional, and obtaining their information from charities, the Internet and the media.

Our participants discussed how their cancer diagnosis had prompted them to think about dietary changes. This may reflect a desire to take control or have some sense of agency post‐diagnosis (Kassianos, Coyle, & Raats, 2015), and is also consistent with the idea that a cancer diagnosis may be a “teachable moment”, in which individuals are motivated to adopt health behaviours (McBride & Ostroff, 2003). However, this hypothesis is at odds with population studies which have found little evidence of sustained positive health behaviour changes as a result of a cancer diagnosis (Kim et al., 2013; Milliron, Vitolins, & Tooze, 2014; Williams, Steptoe, & Wardle, 2013). This may be because patients are not given the tools (advice, support) to realise the potential of the “teachable moment”, or have other competing interests that take priority over dietary change. However, it also suggests people may be overestimating the extent to which they have made lifestyle changes. This is consistent with findings that people think that their behaviours are already good (Anderson, Steele, & Coyle, 2013; Dowswell et al., 2012; Satia et al., 2009) and on the whole they report continuing these post‐diagnosis (Satia et al., 2009).

Our participants reported making changes that included trying to follow a “healthy balanced diet”, reducing specific foods (e.g. high fat foods, red meat) and increasing specific foods (e.g. fruit). Although participants reported that their cancer diagnosis had prompted them to make these positive dietary changes, the motivations for doing this seemed to be driven by beliefs about the importance of diet for improving general health rather than cancer‐related (e.g. to reduce their risk of recurrence). This is consistent with other studies that have shown general health to be an important motivation for healthy eating in adults, and particularly for older adults (Dijkstra, Neter, Brouwer, Huisman, & Visser, 2014). It is also not unreasonable that cancer outcomes are not a key driver for dietary change given that there is not yet evidence that diet quality has a direct influence on outcome for all cancers. In addition, although some studies have found evidence for an effect of diet on overall morbidity (Chlebowski et al., 2006), the precise mechanisms are not yet understood, and the relationship may well be indirect, for example through the role of diet in overall energy balance.

The fact that cancer was not mentioned in relation to motivations for eating healthily also suggests cancer survivors may not be aware of the added benefits of a healthy diet after a cancer diagnosis—because they are at increased risk of conditions associated with lifestyle (Brown et al., 1993; Travis et al., 2006). Public awareness of the link between some aspects of lifestyle and cancer is known to be low (Redeker, Wardle, Wilder, Hiom, & Miles, 2009) and the same may be true for cancer survivors. On the other hand, it may simply be that post‐diagnosis, individuals are more driven to change their diet by a positive (feel good) approach as opposed to a preventive (don't get sick) approach. Focusing on associations between diet‐ and cancer‐specific outcomes may result in feelings of blame, personal guilt or responsibility at one's cancer diagnosis or recurrence (Bell, 2010), which patients may seek to avoid. There is some evidence that interventions seeking to change diet can have a positive impact on quality of life (Kassianos, Coyle, & Raats, 2015). Interventions framed in terms of the potential benefits of a healthy diet for overall well‐being and quality of life may be attended to more than those focused on risk reduction.

Recommendations have been produced that suggest cancer survivors should receive lifestyle counselling (Murphy & Girot, 2013; Travis, Demark Wahnefried, Allan, Wood, & Ng, 2013; WCRF and AICR, 2007). However, our participants did not recall receiving professional advice about diet. This may be because such information was provided, but at a time when patients were too burdened or overloaded to take the advice on board, and therefore do not recall it (James‐Martin, Kockwaza, Smith, & Miller, 2013). However, surveys also indicate that many health professionals do not discuss lifestyle change with their patients (Anderson, Caswell, Wells, & Steele, 2013; Daley et al., 2008; Macmillan Cancer Support/ICM, 2011), and a recent survey of oncology health professionals found that only half were aware of dietary guidelines for cancer survivors (Williams, Beeken, Fisher, & Wardle, 2015). Lack of guidelines, the belief that diet would not affect cancer outcomes and not being the right person to give advice were all identified as barriers to providing dietary advice (Williams et al., 2015). In line with previous research (Kassianos, Coyle, et al., 2015), our participants placed importance upon receiving health professional advice on diet, but found the advice they did receive insufficient, and at times inconsistent. There is therefore a potential need to support health professionals to locate the current guidelines for cancer survivors, to understand the evidence base with respect to long‐term outcomes and to recognise how their role may be important for the delivery of this advice to patients.

With the lack of health professional advice, our participants reported seeking and obtaining dietary information from the Internet and media. This was also found in a recent qualitative study of prostate cancer survivors' perceived influences on dietary change (Kassianos, Coyle, et al., 2015). Use of informal sources may in part explain why some of our participants' beliefs about diet were less well established (e.g. white flour, sugar and food in polystyrene containers being harmful; manuka honey and dietary supplements being beneficial) (WCRF and AICR, 2007). Dietary advice is poorly reported in the media (Cooper, Lee, Goldacre, & Sanders, 2011; Goldacre, 2009); up to two‐thirds of dietary health claims made in UK newspapers have been shown to have insufficient evidence to support them, especially in tabloid newspapers (Cooper et al., 2011). Although our participants were able to find some reliable sources of information on charity websites, they reported difficulties in knowing what to believe. Health professionals could provide guidance in this area that would be welcomed.

This study had a number of limitations. Although the evidence for an association between dietary factors and cancer is stronger for certain types of cancers (e.g. red and processed meat and colorectal cancer), views and advice received by participants in our study did not appear to vary based on their cancer type. However, given the small and heterogeneous sample, it is difficult to draw firm conclusions about the absence or presence of any patterns based on participant characteristics, and we were not seeking to do so. Our sample included people with various types of cancers, treatments and time since diagnosis, making it difficult to compare beliefs and experiences, although it gives a good overview of cancer survivors' views. Results from this study must be interpreted within the limitations of the sample. All participants were White British, relatively young, and well educated. Previous research has identified different drivers for healthy eating in older adults and those from lower socio‐economic groups. Although not apparent in our study, gender and the role of partners and other family members, may also be important (Kassianos, Coyle, et al., 2015; Mroz & Robertson, 2015). Future research should seek to explore this in more depth.

Furthermore, self‐selection bias could explain the generally positive responses to dietary change in the current study. Those interested in our study may be those with a long‐term interest in healthy lifestyles, or those who have become interested since diagnosis. We did not ask about pre‐diagnosis dietary habits except in the context of how things had changed post‐diagnosis. Bias may also have been introduced by the interviewers; 2, health psychologists and a dietitian. Participants' awareness of these roles may have encouraged answers that were positive about healthy eating, although it was emphasised that there were no right or wrong answers. We recruited partly through an Internet forum meaning that some participants may be particularly motivated to find out information about their cancer, and may have higher Internet literacy. However, we balanced this out by putting up posters in a cancer centre where people were attending routine appointments.

## 5 Conclusions

In conclusion, our findings suggest that cancer survivors are aware of some dietary messages, such as to eat a balanced diet, and report making dietary changes. Although often prompted by a cancer diagnosis, these changes are made primarily because of a desire to feel well and be healthy generally, rather than specifically for disease prevention. The majority of patients' information about diet had been obtained from informal sources (e.g. online, media, others) and there was some confusion over what constitutes a balanced diet. Patients would welcome guidance from health professionals on diet. Interventions that provide clear dietary recommendations for those diagnosed with cancer, and which emphasise the benefits of healthy eating for overall well‐being, may be particularly well‐received. Future research should seek to explore how best to support health professionals to provide this advice.

## Competing interests

The authors declare that they have no competing interests.

## Authors' contributions

HC and JW conceived of the study. All authors contributed to the development of the topic guide. HC, KW, and RB conducted the qualitative interviews. HC, KW, and RB analysed the qualitative interviews in NVivo and generated the list of themes. All authors helped draft the manuscript. All authors read and approved the final manuscript.
